# Stromal Changes are Associated with High P4HA2 Expression in Ductal Carcinoma in Situ of the Breast

**DOI:** 10.1007/s10911-021-09504-4

**Published:** 2022-01-25

**Authors:** Marie Colombe Agahozo, Mieke van Bockstal, Pieter J. Westenend, Christine Galant, Kathleen Lambein, Emily Reisenbichler, Renata Sinke, Serena Wong, Carolien H. M. van Deurzen

**Affiliations:** 1 Department of Pathology, Erasmus MC Cancer Institute, Rotterdam, the Netherlands; 2 Faculty of Medicine and Dentistry, Université Catholique de Louvain, Ottignies-Louvain-la-Neuve, Belgium; 3 Laboratory for Pathology Dordrecht, Dordrecht, The Netherlands; 4 Department of Pathology, AZ Sint-Lucas Hospital Ghent, Ghent, Belgium; 5 Department of Pathology, Yale School of Medicine, New Haven, USA; 6 Pathan, Rotterdam, The Netherlands

**Keywords:** Breast ductal carcinoma in situ, Stromal changes, P4HA2 expression, Ipsilateral recurrence

## Abstract

Ductal carcinoma in situ (DCIS) of the breast is able to induce stromal changes, which likely reflect the crosstalk between DCIS and its microenvironment. These changes harbor prognostic information, although the interobserver variability of scoring stromal changes is moderate. A more robust evaluation of the DCIS-associated stroma is therefore needed. The aim of this study was to characterize P4HA2 expression, which is involved in collagen biosynthesis, in DCIS and to assess whether P4HA2 expression enables a more robust evaluation of the DCIS-associated stroma compared to histomorphology. This study included 410 patients with DCIS. Stromal changes were scored on hematoxylin/eosin-stained whole slides. P4HA2 expression in DCIS-associated stroma was assessed by whole slide immunohistochemistry. One hundred DCIS lesions were evaluated by seven pathologists to study the interobserver variability in the assessment of stromal changes and stromal P4HA2 expression. High P4HA2 expression in stromal fibroblasts was present in 14.1% of the patients. High P4HA2 expression was associated with the presence of periductal stromal changes (P = 0.004). The interobserver variability was similar for the assessment of stromal changes and the percentage of P4HA2-positive fibroblasts. Although we demonstrated a significant association between high P4HA2 expression in fibroblasts and the morphological presence of stromal changes, it seems unlikely that P4HA2 expression can be used as an alternative for the histopathological evaluation of the DCIS-associated stroma.

**Supplementary Information:**

The online version contains supplementary material available at 10.1007/s10911-021-09504-4.

## Background

The detection rate of ductal carcinoma in situ (DCIS) of the breast has dramatically increased over the last decades, due to the implementation of mammographic screening [1–3]. It is a heterogeneous disease with a diverse biological behavior [4, 5]. When left untreated, a proportion of DCIS cases will progress into invasive carcinoma, while others remain indolent. However, there are no reliable biomarkers to predict which cases will progress. Therefore, the majority of patients is treated with surgery, with or without adjuvant radiotherapy [5–7]. This means that a substantial proportion of patients is overtreated, leading to unnecessary costs and morbidity [8]. Currently, several DCIS characteristics, including nuclear grade and the presence of comedonecrosis, are routinely mentioned in histopathological reports, since these characteristics are deemed to be associated with DCIS behavior [6]. Additionally, surrogate molecular subtypes based on the expression of estrogen receptor (ER), progesterone receptor (PR) and human epidermal growth factor receptor 2 (HER2) by immunohistochemistry were also suggested to distinguish the biological behavior of DCIS [5, 9, 10]. Nevertheless, DCIS progression remains a poorly understood process.

DCIS is able to induce stromal changes, which have been associated with DCIS behavior [11–14]. More specifically, myxoid stroma is associated with ipsilateral recurrence and sclerotic stroma is associated with signs of DCIS regression [12, 13]. DCIS regression is regarded as a multistep process which starts with periductal stromal changes that indulge the neoplastic cells, whereby the epithelial cells shrink or disappear, leaving a scar-like structure [8]. These scar-like structurers are likely composed of collagen fibers. DCIS-associated stromal changes, including DCIS regressive changes, often co-occur with DCIS characteristics that are associated with a poor prognosis and/or aggressive phenotype, such as the HER2-positive subtype and high numbers of tumor infiltrating lymphocytes (TILs) [13]. These DCIS-associated stromal changes demonstrate the importance of the crosstalk between DCIS and its microenvironment, which is likely to be involved in DCIS progression. It is therefore of major importance to understand this interaction. Currently, periductal stromal changes are determined in hematoxylin and eosin (H&E)- stained slides, which is characterized by considerable inter-observer disagreement [15]. Immunohistochemical markers that enable more robust evaluation of the periductal stroma are therefore needed.

Since stromal changes are likely composed of collagen fibers, a candidate marker for periductal could be propyl-4-hydroxyproline α subunit 2 (P4HA2). P4HA2 is one of the three isoforms of the α subunit of the collagen propyl-4-hydroxyproline (P4H) complex, which is a key regulator of the collagen biosynthesis. For proper collagen folding, P4H catalyzes the hydroxylation of pro-collagen, whereby the α subunit is required for peptide-substrate binding and enzymatic activation [16]. P4HA2 expression therefore contributes to collagen production through P4H formation. Within invasive breast cancer, P4HA2 gene-expression is significantly upregulated compared to normal breast tissue [17] and high expression has been associated with poor prognosis and metastasis [17, 18]. More recently, stromal P4HA2 expression was reported to be higher in DCIS associated with an invasive component, compared to pure DCIS [19]. High P4HA2 expression in DCIS was associated with shorter local recurrence-free survival. However, Toss et al. did not correlate P4HA2 expression with morphological periductal stromal changes. The aim of this study was therefore to characterize P4HA2 in DCIS and assess whether P4HA2 expression can be used as a positive marker for stromal changes.

## Patients and Methods

### Patient Population

This study included 410 patients with pure DCIS from a previously, well characterized cohort [9] These patients were diagnosed between 2000 and 2016 at the Erasmus Medical Center—Cancer Institute Rotterdam or at the Laboratory for Pathology Dordrecht. Clinical data comprised age at diagnosis and ipsilateral recurrence, which was defined as recurrence of in situ or invasive disease ≥ six months after the initial diagnosis. A central pathology review of all tissue slides was performed to assess DCIS grade, density and position of TILs and presence of periductal stromal changes. TILs density was characterized as high or low, TILs position (excluding those cases with a minimal number of TILs) as periductal, touching or intraductal and stromal changes were characterized as sclerotic or myxoid, as previously described [20]. DCIS surrogate subtypes were based on ER, PR and HER2 expression by immunohistochemistry, as previously described [9]. A cut-off of > 10% nuclear immunoreactivity for ER and PR was applied, according to the Dutch guidelines for hormone receptor status. HER status was evaluated according to the ASCO/CAP guidelines [21, 22]***.*** Informed consent and approval by the local ethics committee were not required for this study, since the Dutch law permits the anonymous use of encoded residual human tissue for scientific purposes [23].

### P4HA2 Expression by Immunohistochemistry

To determine the P4HA2 expression in DCIS-associated stroma, we stained one 4-µm thick formalin-fixed paraffin-embedded (FFPE) whole tissue slides per patient by using an automated immunostainer (Ventana Benchmark ULTRA, Ventana Medical Systems, Arizone, USA). After deparaffination, heat-induced antigen retrieval with CC1 (pH 9.0) was applied for 32 min. The slides were then incubated with P4HA2 monoclonal antibody (Invitrogen, MA5-24,599, clone: CL0351, dilution 1:800) for 32 min at 37˚C. For visualisation, the Optiview kit with amplification (Ventana Medical Systems) was used, followed by a hematoxylin II counter stain for 8 min and a blue coloring reagent for 8 min according to the manufacturers’ instructions (Ventana).

### Scoring of P4HA2 Expression on Whole Slides

Cytoplasmic expression of P4HA2 in DCIS-associated stroma was scored as the total percentage of P4HA2-expressing fibroblasts in the stroma. For dichotomization of the DCIS-associated stroma, a cut-off was set at > 60%, whereby 0–60% was considered as low and > 60% was considered as high P4HA2 expression, adapted from Toss et al. [19]. P4HA2 expression in epithelial and myoepithelial cells were not included in this score. All cases were scored by two observers using a multiheaded microscope, whereby consensus was reached in case of disagreement.

### Assessing Stromal P4HA2 Expression as a Marker for Stromal Changes

Because of the strong association between stromal changes and stromal P4HA2 expression, we assessed whether stromal P4HA2 expression is a more robust marker for periductal stromal changes than histopathological evaluation. For this purpose, the interobserver variability between stromal P4HA2 expression and stromal changes based on hematoxylin/eosin (H&E) stained slides of resection specimens was compared. The most recently diagnosed 100 patients were selected for this interobserver evaluation, including one digitalized H&E stained slide and one corresponding P4HA2 immunohistochemically stained slide per patient. Seven pathologists participated in this study.

**Fig. 1:**
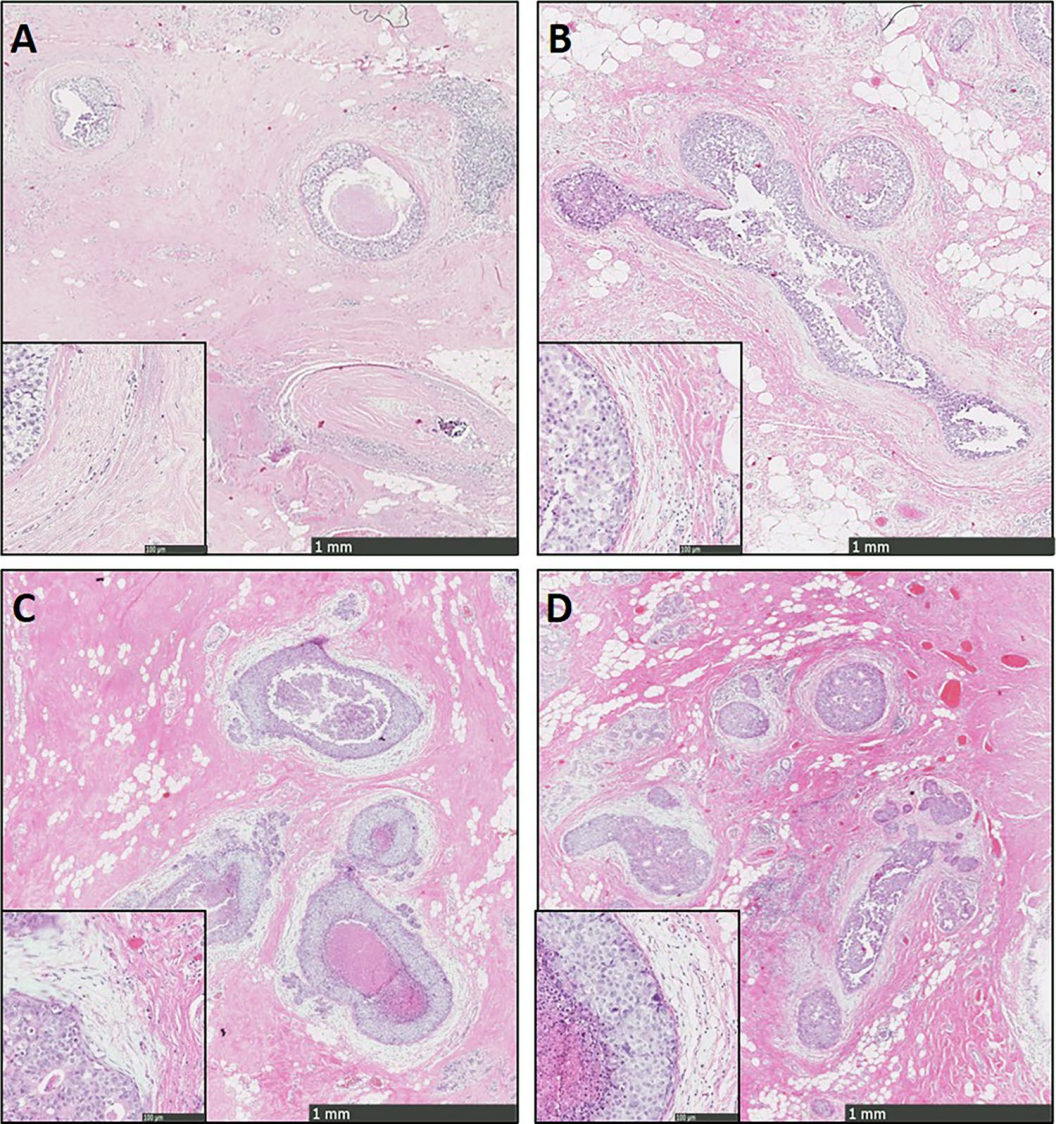
Examples of sclerotic (**A**, **B**) and myxoid (**D**, **E**) stroma at 5 × magnification with 20 × magnification inserts

**Fig. 2:**
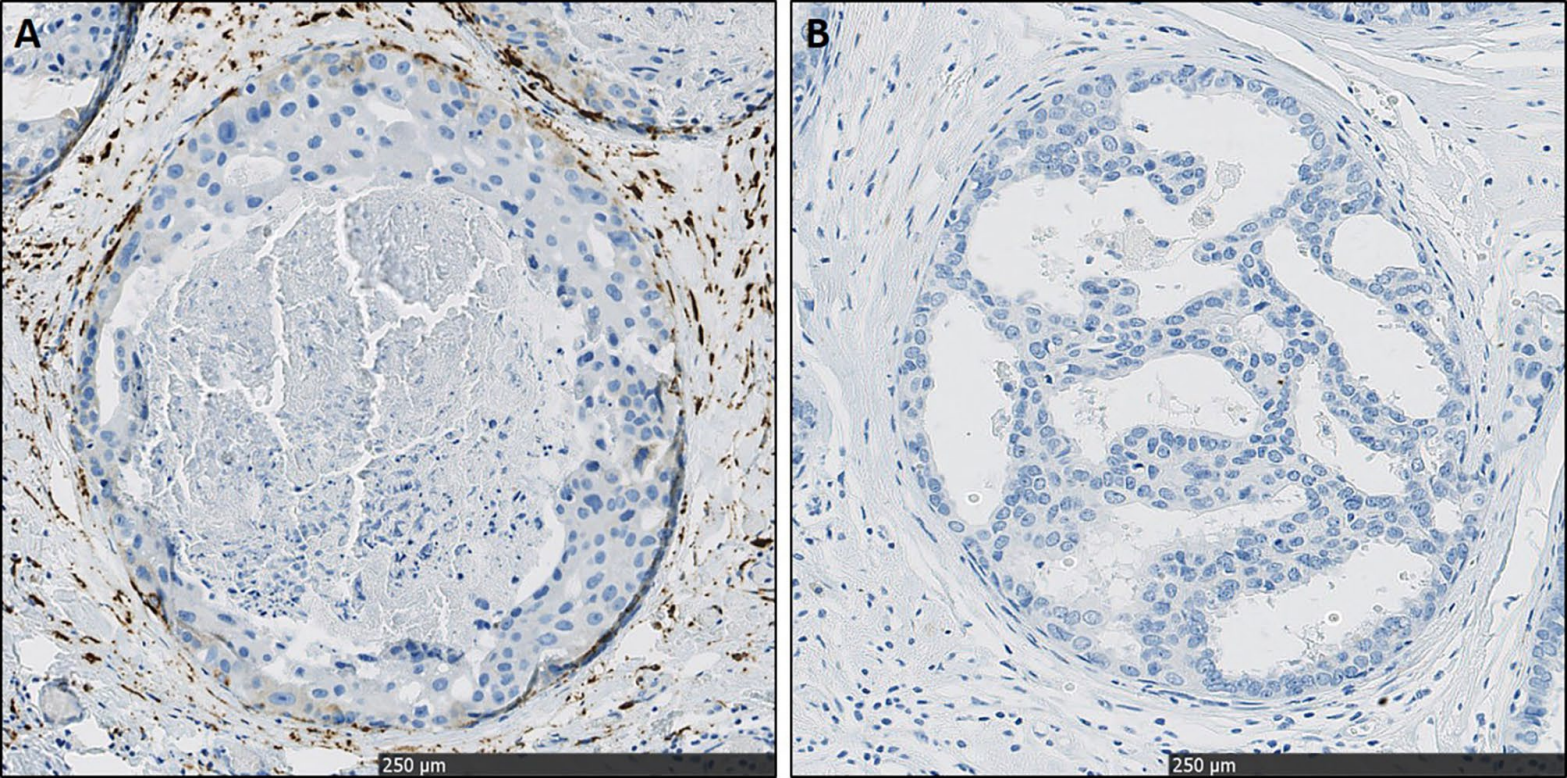
Patterns of cytoplasmatic P4HA2 expression in DCIS-associated stroma, classified as high (**A**) and low (**B**) both at 20 × magnification

Stromal changes were first scored on H&E slides. These changes, sclerotic or myxoid stroma, had to be evidently present in the stroma that directly surrounds the DCIS-affected ducts, by using normal distant stroma as a reference. Focal stromal changes, defined as less than 1 out of 3 DCIS-ducts surrounded by stromal changes, were scored as ‘no stromal changes’. Evident changes comprised stromal changes in more than 1 out of 3 DCIS ducts [15, 24]. Stromal changes were characterized as previously described [20]. Briefly, DCIS-associated stroma was considered as ‘normal’ in case it was identical to the mammary stroma at a distance. Condensation or hyalinization of collagen fibers in the stroma, resulting in the aspect of a dense sclerotic ring around the affected ducts was considered as ‘sclerotic’. Stroma presenting with a loose, greyish or blueish aspect and containing few collagen fibers was scored as ‘myxoid’. Examples of stromal architecture scored on H&E slides are displayed in Fig. 1. Periductal stromal changes were defined as sclerotic and/or myxoid stroma. Stromal P4HA2 expression was scored as the percentage of positive fibroblasts (ranging from 0 to 100%). Examples of stromal P4HA2 expression are displayed in Fig. 2.

Patients were excluded in case two or more participants did not find any DCIS, a single DCIS duct or micro invasion in the slide.

### Statistical Analysis

IBM SPSS statistics 21.0 was used to perform all statistical analyses. Associations between P4HA2 expression and DCIS characteristics were analyzed using the Chi-square test. The Fisher exact test was used in case of a 2 × 2 table. The Mann–Whitney U test was used to compare continuous variables between DCIS with high and low P4HA2 expression. Only variables with significant P-values (< 0.05) were included in the subsequent logistic regression analysis for multivariable testing.

The overall interobserver variability of periductal stromal changes on H&E slides and stromal P4HA2 expression was tested as previously described by Dano et al. [15]. Briefly, the Krippendorff’s alpha (KA) was calculated using the SPSS micro provided by Hayes and Krippendorff (http://afhayes.com/spss-sas-and-r-macros-and-code.html). We only used dichotomous, nominal variables and set the number of bootstrap samples at 10.000. We also calculated the Cohen’s kappa (K) for each participating duo. These values were interpreted according to the Landis and Koch cut-off values [25]. Lastly, we calculated the interclass correlation coefficient (ICC) for the percentage P4HA2 positive stroma assessed as a continuous variable. Besides the use of the previously determined cut-off of 60% [19], we systematically analyzed which post hoc cut-off value had the highest interobserver agreement for stromal P4HA2 expression.

## Results

### Stromal Changes and P4HA2 Expression

In our previous work, histomorphological periductal stromal changes were identified in 136 out of 410 patients. The majority of these changes, 110 out of 136, were classified as sclerotic and the remainder was myxoid. In this cohort, the localization of TILs was only scored in cases with TILs (n = 239). The remaining cases (n = 171) did not have enough TILs to score the location.

In order to analyze stromal P4HA2 expression, we performed whole slide immunohistochemistry. First, we scored P4HA2 expression in DCIS-associated stroma as the % of positive cells. Overall, the median % of P4HA2 expression was 40.0 (range: 0–100) in DCIS-associated stromal fibroblasts. We did not detect any P4HA2 expression in the DCIS-associated stromal fibroblasts in 34 (8.3%) patients. Representative images of P4HA2 expression in DCIS-associated stroma are shown in Fig. 2.

P4HA2 expression was subsequently dichotomized as high and low according to the proposed threshold by Toss et al., with a ≥ 60% as the stromal cut-off [19]. High P4HA2 expression in DCIS-associated stromal fibroblasts was observed in 58 (14.1%) out of 410 patients.

### Stromal Changes and P4HA2 Expression with Regards to DCIS Characteristics

It was previously described that the histomorphological presence of stromal changes is associated with several DCIS characteristics, including high nuclear grade and HER2 overexpression [11–14]. Here, we investigated the association between stromal P4HA2 expression and clinicopathological characteristics of DCIS.

Table 1 summarizes the results of the expression of P4HA2 in DCIS-associated stroma according to DCIS characteristics. There was no association between P4HA2 expression and ER/PR or HER2 status or nuclear grade. In addition, there was no association between stromal P4HA2 expression and the presence of sclerotic or myxoid changes as separate variables. However, we found an association between high P4HA2 expression and the presence of comedonecrosis (P = 0.010), periductal stromal changes (combined, so either stromal or sclerotic; P = 0.004) and TIL position (P = 0.019) in univariable analysis. Only the association with the localization of TILs remained statistically significant after multivariable analysis (P = 0.037).

**Table 1:** The association between P4HA2 expression with DCIS clinicopathological characteristics (n = 410)

	**P4HA2 expression in stroma**		**Univariate P-value**	**Multivariate P-value**
	**High (n = 58)**	**Low (n = 352)**		
**Age at diagnosis (years)**			0.305	-
- **Median (range)**	58.0 (27.0—84.0)	58.0 (27.0–84.0)		
**Diameter (missing n = 52)(in cm)**			0.291	**-**
- **Median (range)**	2.60 (0.10–9.00)	2.15 (0.05–16.0)		
**Growth pattern**			0.662	-
- **Solid**	30	185		
- **Cribriform**	25	134		
- **Micropapillary**	2	27		
- **Papillary**	1	6		
**Grade**			0.129	-
- **Low**	5	38		
- **Intermediate**	14	127		
- **High**	39	187		
**Calcification**			0.874	-
- **Absent**	15	98		
- **Present**	43	254		
**Comedonecrosis**			**0.01**	0.064
- **Absent**	18	175		
- **Present**	40	177		
**Periducal stromal changes**			**0.004**	0.183
**- Absent**	29	245		
**- Present**	29	107		
**IHC DCIS subtype (missing n = 11)**			0.317	**-**
- **ER + PR ± HER2-**	26	195		
- **ER + PR ± HER2 + **	9	68		
- **ER-PR-HER2 + **	16	65		
- **ER-PR-HER2-**	3	17		
**Density of TILs**			0.758	-
**- Low**	42	245		
**- High**	16	107		
TIL position (n = 239*)			**0.019**	**0.037**
**- Periductal**	28	103		
**- Touching**	9	65		
**- Intraductal**	1	33		

*excluding patients with a minimal number of TILs

### P4HA2 Expression and Recurrence

To assess the prognostic value of periductal P4HA2 expression in DCIS, we studied the association between P4HA2 expression and recurrence. Follow-up data was available for 404 out of 410 patients, with a median follow-up time of 103 months (range:24–218 months). Overall, ipsilateral recurrence (including 3 patients with a DCIS recurrence and 12 patients with an invasive recurrence) was observed in 15 out of 404 patients. The median time to recurrence was 98 months, ranging from 16 to 218 months. High P4HA2 expression was only observed in one patient who developed a recurrence. The remaining 14 patients with recurrence had low P4HA2 expression. Overall, P4HA2 expression was not a prognostic marker for ipsilateral recurrence (data not shown).

### Interobserver Agreement for Periductal Stromal Changes Based on H&E

Seven experienced breast cancer pathologists were included to investigate the interobserver variability of stromal changes based on H&E slides from 100 patients. After data processing, a total number of 95 patients were included for interobserver comparison. Two patients were excluded because of micro-invasion and three patients were excluded because participants did not find any DCIS or only a single DCIS duct. We found the lowest absolute agreement when the stroma was scored as sclerotic or myxoid, whereby all participants agreed in 27.4% of the cases. The absolute agreement of periductal stromal changes of ‘any type’, on H&E was 41.1%. Table 2 presents the absolute agreement per scored variable. The KA value for scoring periductal stromal changes ‘any type’ on H&E was 0.482.

**Table 2:** Absolute agreement among participants scoring 95 patients

Variable	Cases n (%)	Absolute agreement n (%)	No agreement n (%)
Stromal Change 'any type'			
- Present	18 (18.9)	39 (41.1)	56 (59.9)
- Absent	21 (22.1)		
Type Stromal architecture			
- None	21 (22.1)	26 (27.4)	69 (72.6)
- Sclerotic	0 (0)		
- Myxoid	5 (5.3)		
Stromal P4HA2 expression			
- High (≥ 60%)	0 (0)	55 (57.9)	40 (42.1)
- Low (< 60%)	55 (57.9)		

We then assessed the individual agreement for each participant’s duo (Supplementary Tables 1 and 2). We found a higher individual agreement when scoring stromal changes (mean K = 0.494, range = 0.359–0.684; Supplementary Table 1) compared to scoring the type of change (mean K = 0.380, range = 0.245–0.538; Supplementary Table 2).

### Stromal P4HA2 as a Marker for Periductal Stromal Changes

The same group of 7 breast cancer pathologists scored stromal P4HA2 expression. We then analyzed the interobserver agreement for P4HA2 expression. Prior dichotomization, the ICC for the percentage of P4HA2 positive stroma was 0.916. We then dichotomized stromal P4HA2 expression using a 60% cut-off, adapted from Toss et al. [19]. The absolute agreement of dichotomized stromal P4HA2 expression was 57.9% (Table 2). The KA value for this cut-off was 0.418. We then systematically investigated which cut-off is associated with the highest agreement (Table 3). We found the highest KA value with a P4HA2 cut-off value of 30%, KA = 0.504, followed by 25% (KA = 0.489), 40% (KA = 0.461) and 20% (KA = 0.4538). We found the lowest KA value in case of a 10% cut-off (KA = 0.374).

**Table 3:** KA values according to stromal P4HA2 cut-off percentages

Cut-off	KA value
10%	0.374
20%	0.454
25%	0.489
30%	0.504
40%	0.462
50%	0.438
60%	0.418

**Fig. 3:**
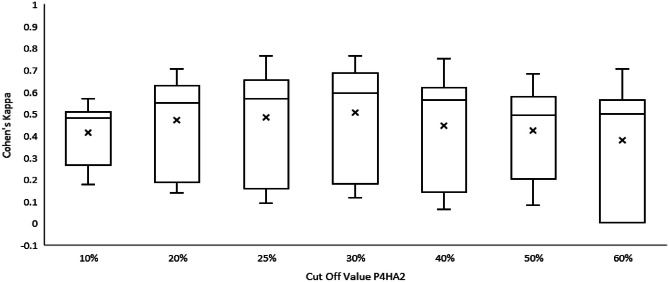
Differences in the distribution in Cohen’s kappa values per pathologist duo according to different P4HA2 cut-off values

Next, we analyzed the individual agreement between each participant’s duos (Supplementary Tables 3, 4, 5, 6, 7, 8 and 9). In agreement with the KA values, we found the highest individual agreement with a cut-off of 30% (mean K = 0.498, range = 0.116–0.762). This cut-off of 30% was also associated with the presence of stromal changes ‘any type’, p = 0.007. Supplementary Table 10 summarizes the results of the expression of P4HA2 in DCIS-associated stroma using a 30% cut-of value, according to DCIS characteristics We observed the lowest individual agreement between participants when scoring stromal P4HA2, with a cut-off of 60%, mean K = 0.377. Of note, one participant (P4) did not agree with any of the other participants in case of stromal P4HA2 expression with a cut-off of 60% (K = 0 for all participants), since P4 rated all DCIS cases as having less than 60% of stromal P4HA2 expression. Detailed data are visualized in Fig. 3.

## Discussion

The biological behavior of DCIS is not well understood. While a proportion of DCIS cases progresses into invasive disease, others remain indolent when left untreated [26–29]. Several DCIS characteristics, including surrogate molecular subtypes, have been suggested as prognostic markers. These do not generally involve the DCIS microenvironment. However, the presence of periductal stromal changes is also associated with DCIS outcome, including ipsilateral recurrence [11–13]. Unfortunately, histopathological assessment of periductal stromal changes is subject to substantial inter-observer variability. Therefore, additional immunohistochemical markers are required to render the diagnosis of periductal stromal changes more robust. P4HA2 expression, which is involved in collagen biosynthesis, was also recently reported to be associated with ipsilateral recurrence in DCIS. Stromal P4HA2 expression might therefore be used as a marker for periductal stromal changes. In this study, we investigated their mutual interrelationship.

We observed cytoplasmic P4HA2 expression in stromal fibroblasts, although we cannot exclude that part of the expression included other cells than fibroblasts. High P4HA2 expression rates in our study are lower than those previously reported by Toss et al. [19]. In that study, a tissue microarray was used instead of whole tissue slides, which could explain the different positivity rates. High P4HA2 expression was associated with high-risk features, including high nuclear grade, ER-HER2 + subtype and the presence of comedonecrosis. Furthermore, high P4HA2 was associated with the presence of stromal changes. These findings indicate that P4HA2 expression could be used as a prognostic factor, which is in line with previous reports [17, 19]. In our cohort, we did not observe a significant association between high P4HA2 expression and increased risk of ipsilateral recurrence, which could be related to the low numbers of recurrences or the follow-up time. Although there was a significance association between P4HA2 expression and stromal changes, only half of the patients with stromal changes also had high P4HA2 expression. Therefore, other factors, like matrix metalloproteinasen, cathepsines or cytokines like TGF-beta and bFGF, might play a more important role in stromal remodeling.

We also assessed the use of stromal P4HA2 expression as a potential alternative for scoring stromal changes. We used the previously determined cut-off for high stromal P4HA2 expression of 60% to dichotomize stromal P4HA2 expression and showed that P4HA2 expression is indeed associated with stromal changes. However, we also demonstrated that the highest agreement was at the 30% cut-off, which also remained significantly associated with the presence of ‘any type’ stromal change. Our findings were comparable to the ones previously reported by Dano et al. [15]. However, the interobserver variability of both variables was similar, suggesting that stromal P4HA2 expression is not a good alternative to optimize the evaluation of stromal changes in DCIS. We therefore do not recommend using stromal P4HA2 expression as a marker for stromal changes. Digital, automatic scoring algorithms or training for pathologists could partly overcome this subjectivity.

This is the first study that evaluated the expression of P4HA2 expression in DCIS on whole tissue slides in a large cohort. The presence of high P4HA2 in DCIS-associated stromal fibroblasts is associated with the histomorphological presence of stromal changes and adverse DCIS characteristics. The scoring of stromal changes and stromal P4HA2 expression had a similar, moderate interobserver variability. Therefore, P4HA2 expression cannot be used as an alternative marker to optimize the evaluation of DCIS-associated stroma, despite its prominent role in stromal collagen synthesis.

## Supplementary Information

Below is the link to the electronic supplementary material.

Supplementary file1 (XLSX 36 KB)
